# Healthcare Provider Perspectives on Pediatric Concussion: The Importance of Formalized Systems of Communication Across Settings

**DOI:** 10.1111/josh.70010

**Published:** 2025-04-21

**Authors:** Doug Gomez, Jody Slocumb, Melissa McCart, Gerard A. Gioia, Deanne Unruh, Julie Haarbauer‐Krupa, Ann Glang

**Affiliations:** ^1^ Department of Psychology University of Oregon Eugene Oregon USA; ^2^ Center for Brain Injury Research and Training University of Oregon Eugene Oregon USA; ^3^ Department of Hospital Medicine Children's National Hospital Washington DC USA; ^4^ Division of Pediatric Neuropsychology Children's National Medical Center Rockville Maryland USA; ^5^ College of Education University of Oregon Eugene Oregon USA; ^6^ Secondary Special Education and Transition University of Oregon Eugene Oregon USA; ^7^ National Center for Injury Prevention and Control Centers for Disease Control and Prevention Atlanta Georgia USA

**Keywords:** brain Injury, concussion, health personnel, pediatric, school health services, students

## Abstract

**Background:**

Healthcare providers play a critical role in the return to school (RTS) process after a child sustains a mild traumatic brain injury (mTBI). The purpose of this study was to examine healthcare providers' perspectives on effective communication with school personnel and gaps within those practices, using a qualitative approach.

**Methods:**

Twelve community‐based healthcare providers in suburban Oregon and Ohio completed semi‐structured interviews between February 2021 and July 2021 via Zoom. Data were analyzed using thematic analysis.

**Results:**

Primary themes centered on how breakdowns in communication between healthcare and education systems occur easily without formalized systems, and how formalized systems of care are beneficial.

**Conclusions:**

Children with mTBI benefit when there is (1) a consistent communication system between school‐based staff, caregivers, and healthcare providers and (2) a clear point person in the school system.

**Implications for School Health Policy, Practice, and Equity:**

Schools should create intentional and formalized communication pathways with healthcare providers as an effective approach to meeting the needs of students and their families.

## 1 Introduction

Traumatic brain injury (TBI) is a significant public health concern, and mild TBI (mTBI) or concussion is the most common presentation [1]. Following mTBI in a child, healthcare providers and school professionals share responsibility for management [2]. After initial care is provided in the healthcare setting, the role of healthcare providers is to monitor symptoms and guide the gradual return to school (RTS) process and activities [3, 4]. Many children with mTBI experience symptoms (e.g., headache, fatigue, light sensitivity, difficulty concentrating, decreased emotional tolerance) that can impact school performance [5], requiring academic accommodations. A return to school that is not appropriately managed by healthcare providers and then progressively managed within the school system can lead to a significant exacerbation of mTBI symptoms [3]. Students in disadvantaged situations, for example, living in low‐income communities, experience higher rates of mTBI [6, 7], have less access to services, and thus require support from schools [8, 9, 10, 11].

To facilitate the creation of an effective school support program, the healthcare‐to‐school link is critical [2, 12]. When healthcare providers and school professionals communicate about the child's symptom profile and support needs, the student is more likely to receive appropriate supports and accommodations at school [3, 12, 13]. Partnerships between school staff and community healthcare personnel are even more important for disadvantaged students and those in poverty given their lack of access to resources outside of the school and healthcare systems [2].

A number of recent reviews illustrate significant gaps in communication between healthcare providers and education professionals who support children with mTBI [14, 15]. Often there is either poor or no communication between healthcare providers and school personnel [16], which can lead to delays in school staff implementing appropriate school supports [17, 18]. The child's caregivers can support the child's RTS process and activity by serving as a link between educators and health care providers. This assumes, however, caregivers are knowledgeable about the effects of mTBI on learning and behavior [19, 20], and have the time to coordinate efforts for school supports. That assumption may be unrealistic for caregivers since concussion and mTBI are unexpected events, and a lack of awareness about the potential effects of mTBI could lead to miscommunication and a lack of school supports [17].

To date, there is little research on the topic of healthcare‐school collaboration in the RTS process. This exploratory qualitative study reports on the findings of interviews conducted with a sample of healthcare providers in suburban Oregon and Ohio between February and July 2021 on their experiences of caring for children with mTBI and their interactions with schools. Specifically, this study addressed the following questions:

1. What communication occurs between healthcare providers and school personnel related to the RTS process?
2. What communication gaps exist between healthcare providers and school personnel related to the RTS process?

## 2 Methods

### 2.1 Participants

Researchers conducted interviews with 12 healthcare providers, 6 each from suburban Oregon and suburban Ohio. Both purposive and convenience sampling were used to select participants who were likely to come in contact with individuals who had sustained an mTBI. Participants were recruited between February 2021 and July 2021 through contacts in medical facilities, project advisors, and through social media. Researchers provided information about the study and contact information for the principal investigator and research assistants.

### 2.2 Instrumentation

Interviewers followed a semi‐structured protocol (Appendix A) to focus on specific topics, while allowing for greater flexibility in the types of follow‐up questions that could be asked. The semi‐structured interview protocol was created by three authors (DG, DU, and AG) with expertise in mTBI and qualitative research, and the protocol consisted of questions to ascertain the structures and procedures in place that facilitate a successful RTS process (Appendix A). The protocol also served to determine what effect the COVID‐19 pandemic had on these procedures [21].

For all interviews, we described the purpose of the project and conducted the interviews as prescribed in IRB‐approved protocols. The interviews were audio‐recorded and transcribed verbatim, removing names to protect the identities of the participants. Initially, we set a goal of interviewing 12 participants, consistent with Guest et al.'s recommendations [22] on sample size needed for thematic saturation, or the point at which new themes are not being identified. We started to reach a point of saturation in data after completing 10 interviews; however, we completed the remaining two interviews and noted that the final two interviews produced detailed examples of sub‐themes.

### 2.3 Procedure

While general RTS models and frameworks have been established, there is very little research on healthcare and school collaboration processes. We took an exploratory qualitative approach to examine this specific aspect of the RTS experience. Exploratory qualitative approaches are recommended when there is a risk of narrowing the field of inquiry before researchers understand the factors involved and the questions that are central to the needs of the target population [23]. This qualitative study was part of a larger quantitative study evaluating the effectiveness of an established RTS model for students with TBI [24].

The healthcare provider interviews were conducted via Zoom to follow COVID‐19 pandemic‐related remote work protocols and scheduled at times convenient for participants. Each participant was compensated $50 for their participation. Interviews lasted between 30 min and 1 h and were facilitated by a qualitative researcher [*author initials removed for anonymity*] or two school psychology graduate students trained in qualitative methods. An experienced qualitative researcher monitored all interviews for fidelity to interview protocol.

### 2.4 Data Analysis

All of the interview data for this study were analyzed using the tenets of Thematic Analysis [25, 26]. Three key decisions were made before the start of the analysis: (1) we analyzed the entirety of the data; (2) we created and defined a set of unique semantic themes using an iterative process of careful reading; and (3) we used a critical realist epistemology to frame the entirety of the study. In this case, applying a critical realist epistemology allowed us to recognize that the healthcare providers who we interviewed were able to describe their personal experiences related to RTS, but we could also recognize that those realities are socially constructed and can be critically examined.

To begin the analysis, interviews were transcribed and then a qualitative researcher and senior research assistant [*author initials removed for anonymity*] read through and coded each interview. Initial interviews were coded together to reach consensus on the different categories. Codes such as “Positive Communication,” “Linkages,” and “Effective Communication” were stored in Dedoose [27], a qualitative analysis software. After the first five interviews, the remaining interviews were coded individually and then discussed in a weekly meeting to again achieve consensus on the coding categories. After all of the coding was complete, both researchers met to discuss potential themes within the codes. The codes were individually analyzed to determine whether the themes were represented well in the data. The qualitative researcher and senior research assistant then met after 1 week to come to a full consensus on the themes and sub‐themes. The next step was to present the themes to the larger research team, comprised of all of the authors of this study. The larger research team then discussed their perspectives on themes and codes, and worked on formalizing and defining the themes. The result of that meeting was the description and definition of each of the themes and sub‐themes. An example of the theme creation process was that we identified there were many similar statements coded as “positive communication,” “linkages,” and “effective communication” that related to the roles of caregivers in RTS. We then observed that within those statements there were also contradictory statements providing examples of breakdowns in communication and warning against relying strictly on caregivers alone for effective communication. This led to the creation of the “breakdowns in communication” theme and a “caregiver communication” sub‐theme.

Throughout the analysis, we adhered to Brantlinger et al.'s [28] best practices to improve on trustworthiness and credibility within a qualitative research project. These practices included triangulation as investigators through our coding practices, analyzing the data collaboratively, discussing the analysis with colleagues on the research team, and documenting each step of the analysis process. These practices provided a helpful framework, which is consistent with the focus on “trustworthiness” and “dependability” discussed by Braun and Clarke [29].

## 3 Results

### 3.1 Participant Characteristics

Participants included 12 healthcare providers. Providers consisted of three pediatricians, two emergency room physicians, two nurse practitioners, two physician assistants, one hospital‐based physician, one physiatrist, and one neurologist (Table 1). Providers identified as mostly female (66%) and white (83%). Demographic information for participants is detailed in Table 1.

**TABLE 1:** Demographic characteristics by site.

	Oregon (*N* = 6)		Ohio (*N* = 6)	
Gender (*n*, %)				
Male	2	33%	2	33%
Female	4	67%	4	67%
Ethnicity (*n*, %)				
Non‐Hispanic	5	83%	6	100%
Missing data	1	17%	0	0.0
Race (*n*, %)				
White	5	83%	5	83%
Asian	0	0.0	1	17%
Missing data	1	17%	0	%
Community medical provider role (*n*, %)				
Pediatrician	2	33%	1	14%
Emergency room physician	1	17%	1	14%
Hospital based physician	0	0.0	1	14%
Physiatrist	1	17%	0	0
Physician assistant	2	33%	0	0
Nurse practitioner	0	0	2	33%
Neurologist	0	0	1	17%

### 3.2 Summary of Results

We found that healthcare providers described communication between themselves and educators as an important component of a positive RTS process for children. We organized the data based on two primary themes, each with a more specific sub‐theme. The first theme was (1) Formalized systems of communication between healthcare providers and educators are beneficial. The sub‐theme was (1a) The best individuals to communicate with within a school for healthcare providers are school nurses, athletic trainers, or school counselors. The second overarching theme was (2) Breakdowns in communication occur easily when there is no formalized system of communication. This theme had a significant sub‐theme, which was (2a) Caregiver involvement is important, but it is not recommended that caregivers serve as liaisons between healthcare providers and educators.

#### 3.2.1 Formalized Communication Systems Are Beneficial

The majority of the healthcare providers interviewed for this study described the benefits of having a formalized system of communication with school staff. Many were part of a formalized communication system within local schools. This Oregon physician described the benefits of a formalized system involving school staff who had expertise in mTBI:

They really help us because they understand the medical side of things and they know what we have the capability of doing and what we try to do to set the stage. And then, they also understand the academic setting and what is and is not possible, and they kind of help bring those two worlds together.

Other benefits included improved relationships between caregivers and educators and an increased likelihood of the school providing accommodations for a child during the RTS process. Additionally, some healthcare providers described how to create formalized systems and the benefits of these systems over time:

And so, we get out there and we say look, you know, bring the school districts to us. So, we have a meeting with the people who deal with this at the school, and all the independent schools do for that matter. You know, to say look, let's develop a city‐wide protocol or at least have a baseline understanding that is common to all of us about what we should be doing when these kids go back [to school].

Several of the providers were not a part of these systems of communication in their areas, but statements such as this one showed an understanding of the potential benefits of creating better linkages between schools and themselves as healthcare providers:

I really don't have any insight to the school part of it, other than what the patient and the family tells me, so that's definitely a big barrier…I think now because I would have no idea who to even, you know, talk to about that. So, just having like a contact person that I knew that I could reach out to would be good…I put my information on there, so that I'm available to them if they have any questions, but typically I've never had them reach back out to me…

#### 3.2.2 Nurses, Athletic Trainers, and School Counselors

Healthcare providers commented that school nurses, athletic trainers, and school counselors are the most effective links to the education system:

I mean, just communication is the biggest challenge and most of the time, if you can get the, if you can get direct communication… if the counselor can represent the school, and the athletic trainer and if you can get them all into a team meeting the communication is, I think, the biggest key [to success].

Healthcare providers described a number of examples of how their current relationship was facilitated by a nurse, school counselor, or athletic trainer:

There's not a system in most places for that. So what we did to develop that system in [location], it was mostly through the athletic trainer that was working with us. She helped connect us with a school and…so, in that program we had that connection. That connection doesn't just exist, you have to make it happen…I think we're just lucky to have an athletic trainer that took the lead and really helped training happen and did a good job with it, and she helped us navigate with counselors and all the parents and was also really good at communicating with parents too. So we were really lucky to have that situation.

Some of the providers who were interviewed were part of a system that included individuals with titles such as “concussion liaison” or “concussion specialist,” but this sub‐theme showed these roles could be filled by a school nurse, athletic trainer, or school counselor. Healthcare providers reported that these individuals created a significant improvement in communication and set the stage for a more successful RTS process for children who had sustained an mTBI.

#### 3.2.3 Breakdowns in Communication

The vast majority of healthcare providers interviewed, whether they were part of a formalized system of communication or not, described breakdowns in communication due to not having a system in place:

I have found that, sometimes the issue when a parent comes and says, you know, ‘They're not doing anything that you put on the school accommodation form,’ you know, ‘They haven't helped my son or daughter at all,’ it's that there's some sort of hiccup in the communication or some sort of breakdown in the communication.

When no formalized communication system was in place, communication breakdowns sometimes led to confusion in school programming:

You know, when they come back to the office and mom's telling me a story and I'm like, ‘what's happening?’ A lot of times if I investigate those things, it's usually a huge misunderstanding, not accurate information that I was given… ‘Why are you making this kid lift weights, you know…’ and that wasn't what was happening. We could probably do a better job of communicating with the trainers and the coaches and making sure things are happening.

Healthcare providers who are used to writing prescriptions without two‐way communication described the challenges:

I mean, you see them and you say, ‘here's what you need to do.’ And we can write notes in our charts and we send letters and say, ‘okay yeah, kid's fine to come back,’ or ‘they need these accommodations’ etc., but I think it's actually a lot easier said than done. And I think we probably have to build better linkages between me and the school and sometimes I don't get any feedback in terms of how they're doing. Or was it okay, were there things that we should have changed or should not have done on an ongoing basis…You know I find out things were great, which is nice, or maybe things were not so good and there's a bunch of other stuff happening in the background…I think better ways to create linkages between the acute care and the rehab and therapists and schools I think, is something that we have to work on.

#### 3.2.4 Caregiver Communication

The healthcare providers in this study noted one of the most problematic patterns in the RTS process is when healthcare providers rely on caregivers alone as the communication link with schools. There were many examples of providers describing these breakdowns in communication. This physician in Ohio was explicit about this process not working within her practice:

I think the approach that doesn't work is tell the kid and the parents to tell the counselor to tell the teachers, or trying to just put it in your note and say, here's the note, give it to the counselor.

Similarly, a pediatrician in Oregon stated, ‘I really try and have the school nurses be the ones implementing the concussion plan, I don't want it to be the families implementing it.’ One of the more apt comparisons of this dynamic was to the children's game of telephone: [30]

One of the challenges that sometimes happens is that when kids are seen, for example in the hospital environment, we're not intricately linked you know, with the school districts. And so, here we are, we're saying one thing, now we provide a note to the parents, the parents take it to the school, the school doesn't have any ongoing linkage with the hospital, so now you're sort of playing a game of telephone to try to make sure that they have the information that they need.

Although providers recognized that relying on caregivers alone can create problems with RTS, they also made it clear that it is important to educate caregivers and include them as a part of treatment. This concussion specialist described the ideal mindset for caregivers when it comes to managing the RTS process:

I think sometimes it ends up being laying the foundation with parents as well as the student who has, you know, suffered that concussion for there to be kind of a reasonable understanding of what is possible and what our goal is here… it's not an ‘us versus them.’ It is a, you know, how can we all work together, what can I do at home, what can a Concussion Specialist and her team do in the medical office, what can the educational team do at the school level, and how can all of that come together to support this individual.

## 4 Discussion

The goals of this exploratory qualitative study were to describe what communication occurs between healthcare providers and school professionals when students return to school following mTBI, and to identify potential gaps in current practice between schools and these healthcare providers. Across healthcare providers interviewed, the most consistent theme was the importance of establishing a system for communicating with educators about students as a key component of RTS. Currently, there are at least four states in the US that have formalized RTS systems mandating a set of responses for students returning to school after an mTBI.

Healthcare providers reported that without such a system, caregivers tend to take on the role of liaison between healthcare providers and school staff. Caregivers can play an active role in the RTS process, but participants in this study described that when they are relied upon as the *only* means of communication, breakdowns in communication between healthcare providers and educators are more likely to occur. Previous research with students with more significant disabilities following TBI showed that school personnel depend on communication with healthcare providers to guide them in implementing effective school supports [13]. Having no communication between hospital personnel and school staff significantly decreases the likelihood that educational services will be tailored to the student's specific needs [13].

The two complex systems caring for children after mTBI—healthcare and education—operate with their own purposes, priorities, and jargon, all of which can affect linkages between the two systems [12]. Children benefit when the professionals in both systems have clear pathways for communication to provide coordinated care [31, 32, 33]. A consistent communication system that includes healthcare providers, caregivers, and school personnel can reduce communication barriers and ensure that accurate information is conveyed between the two systems [34]. This can lead to improved relationships between caregivers and educators, as caregivers have faith that the professionals caring for their child are working together to ensure that their child has a successful RTS process [34].

Critical in any communication system is the identification of a school‐based staff member who has a background in TBI and can facilitate communication between school staff and healthcare providers. Identifying a point of contact within a complex school system supports the implementation of a systemic communication plan. The healthcare providers interviewed in this study identified that school nurses, athletic trainers, or school counselors may be the best point people to ensure that the complex communication loop between healthcare providers and schools is maintained. There is not a specific recommendation that this role needs to be filled by a person holding one of the aforementioned positions, but it is clear that there needs to be a consistent primary contact person [35].

It can be challenging to facilitate communication between these complex systems [12]. One recommendation from Gonsoulin [36] is to engage in the use of collaborative decision‐making, making cross‐system practice and resource decisions jointly. Collaborative decision‐making in practice could look like a healthcare provider communicating directly with a school about what symptoms the student is experiencing and working with the school to identify accommodations that would benefit the student. For example, a student's physician might communicate that the student experiences light sensitivity, resulting in the school counselor suggesting that the student be seated away from windows and allowing the use of sunglasses or a hat. This type of information sharing and collaborative decision‐making can lead to more targeted and effective approaches to meeting the needs of students and their families [36].

For context, a good example of an effective communication system for RTS between healthcare providers and educators exists in the Concussion and TBI program in Central Oregon [37]. This system involves a partnership between a school district, a medical center, and an organization that trains individuals within schools about TBI [38]. The school district provides TBI liaisons who have been trained to act as the point of contact between the medical center and the individual schools. These liaisons can relay medical recommendations as well as share concerns that originate from the schools, which are then relayed back to the healthcare professionals in the center. This type of system was formalized for the primary purpose of improving the RTS process, and the idea of using an intermediary at the school district as a point of contact helps to mitigate some of the difficulty of the healthcare system interacting with each individual school directly. Though this system has been effective in Central Oregon, we believe it is a model that is applicable across the country for RTS. It is important to note that these systems can be challenging to develop and involve identifying what resources exist locally including technology to improve communication, developing an educated point of contact in each school or district, and collaborating to create appropriate releases of information across organizations to address The Health Insurance Portability and Accountability Act (HIPAA) or The Family Educational Rights and Privacy Act (FERPA) concerns.

### 4.1 Implications for School Health Policy, Practice, and Equity

This exploratory study presents the perspectives of one sample of healthcare providers involved in facilitating the RTS process for children who have sustained mTBI. The key finding was that systems that include pathways for communication between healthcare and education settings are beneficial, and the disciplines that are best positioned to act as liaisons with healthcare providers are school nurses, athletic trainers, and school counselors. Findings suggest that breakdowns in communication occur easily when there are not formalized systems. One of the more important practices that can create breakdowns in communication is when healthcare providers rely strictly on caregivers to provide communication to schools about accommodations or other aspects of the RTS process.

School services for students with mTBI could be improved through simple changes in policy and practice. These policies provide structure for schools as they support students with mTBI. For example, leadership within a school or school district should identify a point of contact for local healthcare providers to communicate about students who sustain concussion. Policies that are as simple as appointing a point of contact are crucial in under‐resourced communities because, in these communities, children often do not receive timely medical care [6, 7]. Having a point person who oversees the RTS process could greatly benefit these children, who may be misidentified following concussion, contributing to a negative school trajectory [12, 15]. Additionally, schools can provide more education about TBI for all disciplines within the school system. Education about TBI is a great way to build up awareness and interest, which is the first step in the creation of any formalized system to address concerns about the RTS after an mTBI.

### 4.2 Limitations

This study represents the perspectives and experiences of a group of healthcare providers from two specific geographical regions (Central Oregon and Southwestern Ohio). Participants were selected using purposive sampling of individuals within a range of different healthcare specialties who participate in the RTS process, but the information from these interviews may not be generalizable due to potential differences in community demographics, provider demographics and training, capability of follow‐up with providers (e.g., emergency room physician follow up could be much more challenging than following up with a pediatrician), and differing RTS practices across the country. Rather than the cross‐sectional approach utilized in this study, a longitudinal study of healthcare providers who provide care for youth with brain injury would yield valuable insight into effective approaches to communication with educators. Researchers could follow healthcare providers who are just beginning to develop a relationship with their local school districts to further understand the key levers that enhance clear communication.

### 4.3 Conclusion

Creating formalized systems of communication among members of complex healthcare and education systems is crucial in the RTS process. This study showed an example of healthcare providers who recognized the importance of those systems and provided some examples of key participants within the education setting, including school nurses, athletic trainers, and school counselors. Further studies examining these findings across the United States with different provider types and in different settings will help clarify how to best implement and support effective RTS processes and improve mTBI outcomes.

## Ethics Statement

The research was approved by the Institutional Review Board of the University of Oregon IRB # 09142018.017.

## Consent

Prior to participating in interviews, participants provided online informed consent, including consent to record interviews.

## Conflicts of Interest

Melissa McCart is the director of the Center on Brain Injury Research and Training, and Ann Glang is the former director. Both have received federal funding (e.g., Center for Disease Control, National Institute on Disability Independent Living and Rehabilitation Research) and state contracts (e.g., Washington Department of Social and Health Services, Alaska Mental Health Trust) to conduct research and provide services in the area of brain injury. All other authors have no interests to declare.

## Appendix A

### Healthcare Provider Interview Protocol Related to the Return to School Process Following Brain Injury/Concussion

- What experience do you have with brain injury/concussion?
  ∘ Probes: patients they have served, personal or family experience
  ∘ *Only ask the question below if they answer the first question quickly*:
    - What is the typical protocol when a person enters your office or hospital and you suspect that they may have a concussion?
- Tell me about what happens when a youth exits your care after sustaining a concussion.
  ∘ Probes: What communication procedures are in place that facilitate a successful return to school? Linkages with educators? Communication with parents?
  ∘ After you've seen someone for the first time, what happens next in the process?
- What are your experiences communicating with parents about brain injury/concussion?
  ∘ Probes: approaches that worked well, approaches that didn't
  ∘ Probes: Is your ED familiar with any sort of materials and do you use those for follow up?
- What are your experiences communicating with educators about concussion?
  ∘ Probes: approaches that worked well, approaches that didn't
- How do you recommend educators support a child with a concussion when the child returns to school with symptoms?
- Can you describe an example of a youth that you've worked with when things have gone particularly well?
  ∘ Probes: What went well in terms of communication/coordination of care (with other healthcare providers, school staff, athletic personnel, parents)?
  ∘ What types of accommodations seemed to work well?
  ∘ Were there procedures in place in the school that helped (e.g., monitoring protocol, communication plans, etc.)
- How about when things have not gone as well?
  ∘ Probes: What was problematic in terms of communication (with other healthcare providers, school staff, athletic personnel, parents)?
  ∘ Were there gaps in the transition back to the school setting that caused problems (e.g., poor communication, disagreements about whether or not to provide accommodations)
- What primary challenges do you face in coordinating care for a child with concussion?
- What structures or supports would help you overcome those challenges?
  ∘ Probe: What aspects within the school setting would benefit from improvement? How might you do it?

### Questions About COVID‐19‐Related Changes

- COVID‐19 has led to changes in service provision; can you tell me about how any of those changes affect the way that you see students who have sustained a concussion?
- Do you think any of these COVID‐19 related changes of structures (school or medical provider) have affected students' symptoms or recovery? How?
- How are you doing? How have these changes affected your own practice/place of work?

### Concluding Question

- Is there anything I haven't asked you about that you'd like to share with me related to care coordination for youth with concussions?

## References

[1] J. Haarbauer‐Krupa , M. J. Pugh , E. M. Prager , N. Harmon , J. Wolfe , and K. C. Yaffe , “Epidemiology of Chronic Effects of Traumatic Brain Injury,” Journal of Neurotrauma 38 (2021): 3235–3247, 10.1089/neu.2021.0062. 33947273 PMC9122127

[2] H. T. Keenan , A. E. Clark , R. Holubkov , and L. Ewing‐Cobbs , “Changing Healthcare and School Needs in the First Year After Traumatic Brain Injury,” Journal of Head Trauma Rehabilitation 35, no. 1 (2020): E67–E77, 10.1097/HTR.0000000000000499. 31246877 PMC6930363

[3] G. A. Gioia , “Medical‐School Partnership in Guiding Return to School Following Mild Traumatic Brain Injury in Youth,” Journal of Child Neurology 31, no. 1 (2016): 93–108, 10.1177/0883073814555604. 25535055 PMC4636464

[4] K. McAvoy and J. Haarbauer‐Krupa , “What Schools Need to Know About the Centers for Disease Control and Prevention's Guideline on Diagnosis/Management of Mild Traumatic Brain Injury in Children—A Commentary,” Journal of School Health 89, no. 12 (2019): 941–944, 10.1111/josh.12834. 31691286 PMC7111133

[5] A. Lumba‐Brown , K. O. Yeates , K. Sarmiento , et al., “Centers for Disease Control and Prevention Guideline on the Diagnosis and Management of Mild Traumatic Brain Injury Among Children,” JAMA Pediatrics 172, no. 11 (2018): e182853, 10.1001/jamapediatrics.2018.2853. 30193284 PMC7006878

[6] K. A. Kelly , P. D. Patel , S. Salwi , H. N. Lovvorn, III , and R. Naftel , “Socioeconomic Health Disparities in Pediatric Traumatic Brain Injury on a National Level,” Journal of Neurosurgery: Pediatrics 29, no. 3 (2022): 335–341, 10.3171/2021.7.PEDS20820. 34740192

[7] J. K. Yue , N. Krishnan , J. P. Andrews , et al., “Update on Pediatric Mild Traumatic Brain Injury in Rural and Underserved Regions: A Global Perspective,” Journal of Clinical Medicine 12, no. 9 (2023): 3309, 10.3390/jcm12093309. 37176749 PMC10179657

[8] N. Jimenez , V. Harner , M. A. Oliva , L. Lozano , and M. Fuentes , “The Role of Social Determinants of Health in the Receipt of School Services After Traumatic Brain Injury: A Focus Review on Underserved Pediatric Populations,” NeuroRehabilitation 52 (2023): 569–583, 10.3233/NRE-220210. 37125571

[9] O. Ogunmayowa , A. Lozano , A. Hanlon , F. Paige , N. Cook , and C. Baker , “Social Vulnerability and Traumatic Brain Injury Hospitalizations From Sports and Recreation Among Pediatric Patients in the United States,” Annals of Epidemiology 93 (2024): 19–26, 10.1016/j.annepidem.2024.03.002. 38508406

[10] G. Rigney , J. Jo , K. Williams , D. P. Terry , and S. L. Zuckerman , “Parental Factors Associated With Recovery After Mild Traumatic Brain Injury: A Systematic Review,” Journal of Neurotrauma 40, no. 19–20 (2023): 2015–2036, 10.1089/neu.2023.0015. 37212287

[11] M. R. Zonfrillo , J. Haarbauer‐Krupa , J. Wang , et al., “Effect of Parental Education and Household Poverty on Recovery After Traumatic Brain Injury in School‐Aged Children,” Brain Injury 35, no. 11 (2021): 1371–1381, 10.1080/02699052.2021.1972141. 34529550

[12] J. Haarbauer‐Krupa , A. Ciccia , J. Dodd , et al., “Service Delivery in the Healthcare and Educational Systems for Children Following Traumatic Brain Injury: Gaps in Care,” Journal of Head Trauma Rehabilitation 32, no. 6 (2017): 367–377, 10.1097/htr.0000000000000287. 28060211 PMC6027591

[13] A. Glang , B. Todis , C. W. Thomas , D. Hood , G. Bedell , and J. Cockrell , “Return to School Following Childhood TBI: Who Gets Services?,” NeuroRehabilitation 23, no. 6 (2008): 477–486. 19127001

[14] K. Andersson , M. Bellon , and R. Walker , “Parents' Experiences of Their Child's Return to School Following Acquired Brain Injury (ABI): A Systematic Review of Qualitative Studies,” Brain Injury 30, no. 7 (2016): 829–838, 10.3109/02699052.2016.1146963. 27057776

[15] L. R. Hartman , M. Duncanson , S. M. Farahat , and S. Lindsay , “Clinician and Educator Experiences of Facilitating Students' Transition Back to School Following Acquired Brain Injury: A Qualitative Systematic Review,” Brain Injury 29, no. 12 (2015): 1387–1399, 10.3109/02699052.2015.1071431. 26362526

[16] D. Gomez , A. Glang , J. Haarbauer‐Krupa , et al., “Stakeholder Perspectives on Navigating the Pediatric Concussion Experience: Exploring the Needs for Improved Communication Across the Care Continuum,” NeuroRehabilitation 52, no. 4 (2023): 605–612, 10.3233/nre-220220. 37125574 PMC10481243

[17] K. P. Snedaker , J. P. Lundine , A. H. Ciccia , M. N. Haider , and K. H. O'Brien , “Gaps in Concussion Management Across School‐Aged Children,” Brain Injury 36 (2022): 714–721. 35130810 10.1080/02699052.2022.2034954

[18] A. N. Wan and A. S. Nasr , “Return to Learn: An Ethnographic Study of Adolescent Young Adults Returning to School Post‐Concussion,” Journal of Clinical Nursing 30, no. 5–6 (2021): 793–802. 33351994 10.1111/jocn.15617

[19] M. C. Kay , J. K. Register‐Mihalik , C. B. Ford , R. M. Williams , and T. C. Valovich McLeod , “Parents' and Child's Concussion History as Predictors of Parental Attitudes and Knowledge of Concussion Recognition and Response,” Orthopaedic Journal of Sports Medicine 5, no. 12 (2017): 2325967117742370, 10.1177/2325967117742370. 29242807 PMC5724649

[20] T. Rice and R. Curtis , “Parental Knowledge of Concussion: Evaluation of the CDC's “Heads up to Parents” Educational Initiative,” Journal of Safety Research 69 (2019): 85–93, 10.1016/j.jsr.2019.02.007. 31235239 PMC11924245

[21] D. Unruh , D. Gomez , J. Slocumb , et al., “Stakeholder Perspectives on the School Experiences of Students With Traumatic Brain Injury: The Effects of COVID‐19 Pandemic on Service Delivery,” Journal of School Health 93, no. 5 (2023): 378–385, 10.1111/josh.13280. 36394169 PMC10507668

[22] G. Guest , A. Bunce , and L. Johnson , “How Many Interviews Are Enough? An Experiment With Data Saturation and Variability,” Field Methods 18, no. 1 (2006): 59–82, 10.1177/1525822X05279903.

[23] J. W. Creswell and J. C. Báez , 30 Essential Skills for the Qualitative Researcher (Sage Publications, 2020).

[24] D. Anderson , J. M. Gau , L. Beck , et al., “Management of Return to School Following Brain Injury: An Evaluation Model,” International Journal of Educational Research 108 (2021): 1–21, 10.1016/j.ijer.2021.101773. PMC8076871 33927471

[25] V. Braun and V. Clarke , “Using Thematic Analysis in Psychology,” Qualitative Research in Psychology 3, no. 2 (2006): 77–101, 10.1191/1478088706qp063oa.

[26] V. Braun and V. Clarke , “One Size Fits All? What Counts as Quality Practice in (Reflexive) Thematic Analysis?,” Qualitative Research in Psychology 18, no. 3 (2020): 1–25, 10.1080/14780887.2020.1769238.

[27] Dedoose , Web Application for Managing, Analyzing, and Presenting Qualitative and Mixed Method Research Data (SocioCultural Research Consultants, LLC, 2019).

[28] E. Brantlinger , R. Jimenez , J. Klingner , M. Pugach , and V. Richardson , “Qualitative Studies in Special Education,” Exceptional Children 71, no. 2 (2005): 195–207, 10.1177/001440290507100205.

[29] V. Braun and V. Clarke , Successful Qualitative Research: A Practical Guide for Beginners (Sage, 2013).

[30] wikiHow , “How to Play the Telephone Game,” https://www.wikihow.com/Play‐the‐Telephone‐Game.

[31] Centers for Disease Control and Prevention , Report to Congress: The Management of Traumatic Brain Injury in Children (National Center for Injury Prevention and Control; Division of Unintentional Injury Prevention, 2018).

[32] J. S. Patricios , C. L. Ardern , M. D. Hislop , et al., “Implementation of the 2017 Berlin Concussion in Sport Group Consensus Statement in Contact and Collision Sports: A Joint Position Statement From 11 National and International Sports Organisations,” British Journal of Sports Medicine 52, no. 10 (2018): 635–641, 10.1136/bjsports-2018-099079. 29500252 PMC5931244

[33] L. K. Purcell , G. A. Davis , and G. A. Gioia , “What Factors Must Be Considered in ‘Return to School’ Following Concussion and What Strategies or Accommodations Should Be Followed? A Systematic Review,” British Journal of Sports Medicine 53, no. 4 (2019): 250, 10.1136/bjsports-2017-097853. 29500251

[34] G. A. Gioia , “Return to School: When and How Should Return to School Be Organized After a Concussion?,” in Sports Concussions: A Complete Guide to Recovery and Management, ed. I. Gagnon and A. Ptito (CRC Press, 2017), 241–261.

[35] P. Wildgoose , D. Diep , A. Rendely , N. Kuwahara , and J. D. Carson , “Barriers to and Facilitators of Return to Learning Following a Sport‐Related Concussion: Perspectives of Female Secondary School Students,” Canadian Family Physician 68, no. 3 (2022): 203–210. 35292460 10.46747/cfp.6803203 PMC9833201

[36] S. Gonsoulin and N. Read , Improving Educational Outcomes for Youth in the Juvenile Justice and Child Welfare Systems Through Interagency Communication and Collaboration. Practice Guide (National Evaluation and Technical Assistance Center, 2011).

[37] B. Injury , “Concussion & Traumatic Brain Injury (TBI),” High Desert Education Service District, accessed April 4, 2025, https://www.hdesd.org/services/traumatic‐brain‐injury/.

[38] A. Glang , B. Todis , P. Sublette , B. Eagan‐Brown , and M. Vaccaro , “Professional Development in TBI for Educators: The Importance of Context,” Journal of Head Trauma Rehabilitation 25, no. 6 (2010): 426–432. 21076243 10.1097/HTR.0b013e3181fb8f45

